# An Exploratory Study on the Effects of Forest Therapy on Sleep Quality in Patients with Gastrointestinal Tract Cancers

**DOI:** 10.3390/ijerph16142449

**Published:** 2019-07-10

**Authors:** Hyeyun Kim, Yong Won Lee, Hyo Jin Ju, Bong Jin Jang, Yeong In Kim

**Affiliations:** 1 Department of Neurology, Catholic Kwandong University, International St. Mary’s Hospital, Incheon 1600-8291, Korea; 2 Department of Internal Medicine, Catholic Kwandong University, International St. Mary’s Hospital, Incheon 1600-8291, Korea; 3 College of Medicine, Catholic Kwandong University, International St. Mary’s Hospital, Incheon 1600-8291, Korea; 4 Graduate School of Healthcare Convergence, Catholic Kwandong University, Incheon 1600-8291, Korea

**Keywords:** forest therapy, cancer, sleep

## Abstract

The improvement of sleep quality in patients with cancer has a positive therapeutic effect on them. However, there are no specific treatment guidelines for treating sleep disturbance in cancer patients. We investigated the effect of forest therapy on the quality of sleep in patients with cancer. This study was conducted on nine patients (one male, eight female; mean age, 53.6 ± 5.8 years) with gastrointestinal tract cancer. All patients participated in forest therapy for six days. They underwent polysomnography (PSG) and answered questionnaires on sleep apnea (STOP BANG), subjective sleep quality (Pittsburgh Sleep Quality Index, PSQI), sleepiness (Stanford and Epworth Sleepiness Scales), and anxiety and depression (Hospital Anxiety and Depression Scale) to evaluate the quality of sleep before and after forest therapy. Sleep efficiency from the PSG results was shown to have increased from 79.6 ± 6.8% before forest therapy to 88.8 ± 4.9% after forest therapy (*p* = 0.027) in those patients, and total sleep time was also increased, from 367.2 ± 33.4 min to 398 ± 33.8 min (*p* = 0.020). There was no significant difference in the STOP BANG score, PSQI scores, daytime sleepiness based on the results of the Stanford and Epworth Sleepiness Scales, and depression and anxiety scores. Based on the results of this study, we suggest that forest therapy may be helpful in improving sleep quality in patients with gastrointestinal cancers.

## 1. Introduction

The natural environment is increasingly recognized as an alternative therapeutic resource for the emotional relaxation and stress management of urban people. The term “forest bathing” was suggested in Japan in 1982. From forest bathing, the therapeutic effects of forest were studied, such as enhancing health and wellness and reducing negative emotions [1]. Recent studies have proposed that “forest therapy” offers benefits like improvement of depression/anxiety symptoms and the reduction of blood pressure due to autonomic nervous system stability [2,3,4]. Forest therapy has also shown health benefits such as lower blood pressure, decreased sympathetic nervous system activity, and increased parasympathetic nervous system activity [2,5]. Forest therapy significantly influences the reduction of stress and emotional relaxation [6,7].

Sleep disturbance is a very common symptom within the general population as society modernizes. Previous studies have shown that short nocturnal sleep time is a risk factor for breast and colorectal cancers [8,9,10]. Sleep disturbance in breast cancer patients was associated with higher all-cause mortality risk in a 30-year follow-up study [11]. In addition, improvement with respect to sleep disturbance was reported to be associated with survival in cancer patients [12]. In particular, decreased nocturnal sleep duration was reported to have a negative impact on cancer survival [13]. However, knowledge about the relationship mechanism between sleep disturbance and cancer outcome is insufficient. Unfortunately, sleep disturbance in cancer patients is associated with many untreatable factors, such as the character of the disease, existing sleep breathing disorders, and psychological symptoms related to cancer and chemotherapy. Therefore, it is difficult to successfully treat sleep disturbances [14]. This study was designed to investigate the effects of forest therapy on cancer patients who were suffering from sleep disturbance.

## 2. Methods

This is a pretest–posttest study design about the effects of forest therapy on patients with gastrointestinal cancer. Among these patients, those who were able to travel for one week and those who agreed to the polysomnography were enrolled in this study. Patients currently undergoing chemotherapy or who underwent cancer-related major surgery less than three months previously were excluded from this study. We provided a personal exercise program to allow for travel for forest therapy during the study period. Before participating in forest therapy, walking exercise for more than three days a week and 30 min or more per day was recommended. Participants received nutritional counseling for cancer healing before the forest therapy program. In addition, quality of sleep, sleepiness, and emotional status before and after forest therapy were evaluated using sleep questionnaires, including the Korean version of the Pittsburgh Sleep Questionnaire Index (PSQI) [15], Insomnia Severity Inventory (ISI) [16], Stanford Sleepiness Scale (SSS) [17], Epworth Sleepiness Scale (ESS) [18], STOP BANG [19] to evaluate sleep apnea, Hospital Anxiety and Depression Scale (HADS) [20], and polysomnography (PSG) for volunteers who suffered from gastrointestinal tract cancers.

**Figure 1:**
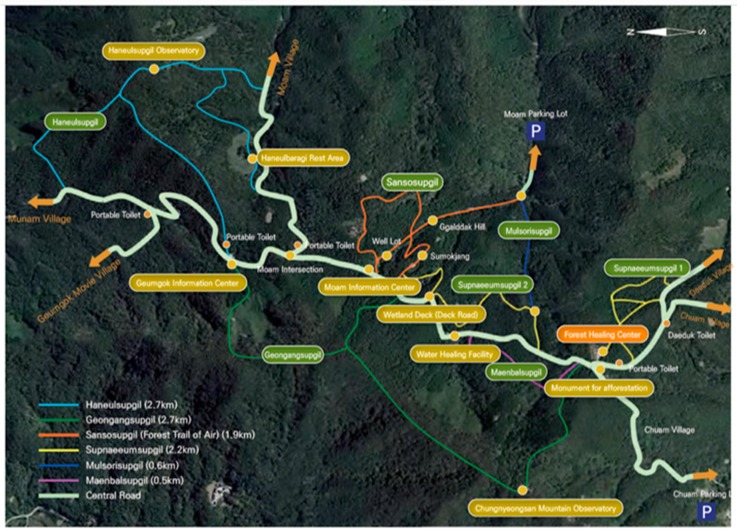
Jang-Seong forest healing area in Chungnyong mountain. It is about 37ha of the forest healing area in Jang-Seong. There is 10.8km forest healing trail. Walking program was provided by choosing an easy-to-walk trail.

**Figure 2:**
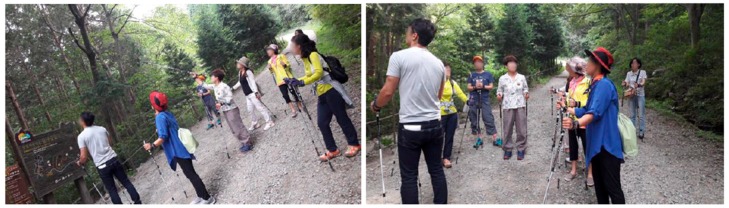
Forest therapy program. Participants were taking a walk in the forest and listening to explanations about tree and forest animals.

Volunteers participated in the forest therapy program by staying at the Jang-Seong forest healing center for 6 days and 5 nights. The Jang-Seong forest activities facility is one of the national centers for forest activities based at Bang-jang Mountain, an outstanding natural location, and Chung-yeon Mountain, where the healing forest in Korea is located (Figure 1). The area is 373 ha with several species of trees, including cypress (42%), Japanese cedar (18%), larch (7%), and others (33%). The patients were advised to maintain a regular sleep and wake time, and were accompanied by regular meal times and exercise programs. During their stay, participants took part in the forest therapy program, which utilizes various environmental factors, such as scenery, phytoncide (antimicrobial volatile organic compounds), and anions. The program consists of recreation, meditation, aroma therapy, Hinoki cypress experiences, stretching exercises in the forest, as well as foot baths in warm water with Hinoki wood pieces. Forest healing recreation took place in groups and lasted about 30 min. The participants then had meditation time of about 40 min on a hammock that hung between the trees. Aromatherapy massages using oil extracted from forest wood and the experience of wood sculpting were provided to the participants. Patients participated in these activities each day, and their schedules varied from day to day (Figure 2).

This study design was approved by the appropriate ethics review board (IS18OISE0025). All patients gave their consent to participate in the study. We analyzed the results from PSG and the sleep questionnaire before and after forest therapy to evaluate changes of sleep quality; we used a paired *t*-test to analyze the results.

## 3. Results

Eleven cancer patients were enrolled in this study. One patient returned home without completing the program due to personal issues. Another patient with advanced stage gastric cancer was unable to complete the program because their disease had worsened. Nine patients participated in all activities provided during the forest therapy program. They were satisfied with the activities, especially meditation and walking through the forest.

In all, nine patients took part in the sleep questionnaire and PSG before and after forest therapy. Eight of the participants were women. The mean age of all patients was 53.9 ± 5.8 years. Mean Body Mass Index (BMI) was 21.9 ± 3.4 kg/m^2^. Their BMI scores did not change before and after forest therapy (Table 1). From the PSG results (Table 2), sleep efficiency was increased from 79.6 ± 6.8% before forest therapy to 88.8 ± 4.9% after forest therapy (*p* = 0.038). Wake after sleep onset (WASO) was decreased from 78.6 ± 25.3 to 44.9 ± 22.2 (*p* = 0.026) after forest therapy. Total sleep time was also increased, but there was no statistically significant difference due to the limitations of the preliminary study (pre-therapy, 373.0 ± 34.8; post-therapy, 405.7 ± 27.9; *p* = 0.097). There was no change in the sleep architecture and proportion of each sleep stage. Periodic limb movements (PLM) during sleep did not change before and after forest therapy. In the sleep questionnaire analysis (Table 3), there was no difference in the STOP BANG score, daytime sleepiness based on the results of the Stanford and Epworth Sleepiness Scales, and depression and anxiety scores.

## 4. Discussion

According to statistics from the Korea Forest Service in 2010, about 63% of the country’s area is made up of forests. However, Korean forests are not suitable for residential environments due to unlivable terrain, and most people live in cities. Forests have abundant resources, and efforts to use and develop them in various ways are ongoing. Research is underway to utilize forests for education and therapeutic purposes for specific populations, as well as for recreation and leisure for the general population. Korea has been experiencing a rapid increase in industrialization and urbanization, while opportunities for people to be exposed to nature have been decreasing [21]. On the other hand, the demand for leisure, sports, and health promotion has increased. In Korea, which is rich in forest resources, efforts to actively utilize forest resources for public health and leisure are increasing in response to these social demands.

This study reported that forest therapy improved the quality of sleep of cancer patients using polysomnography, that is, a gold standard of sleep recording. The results showed that total sleep time increased by about 30 min and efficiency of sleep increased by 8% after forest therapy.

The PSQI for assessing subjective sleep quality was 8.0 ± 4.4 before forest therapy and 7.3 ± 4.9 after forest therapy. A PSQI score of 5 or more indicates poor sleep quality. Participants showed poor quality of sleep before and after forest therapy. After forest therapy, improvement was observed, but it was not statistically significant. More improvement in the sleep index could be expected if a larger number of patients and a longer period of forest therapy were used.

There are several suggested mechanisms that explain the improvement of sleep quality for those cancer patients who took part in forest therapy. Healthy stimulation of the five senses in the forest, forest oils such as phytoncide, circadian-rhythm recovery through a regular sleep–wake cycle, timed exercise, and healthy diet and meals provided during forest therapy could be considered as relevant factors. First, the stimulation of the five senses—sight, smell, touch, hearing, and taste—during forest therapy was rich and healthy for the cancer patients. This could lead to physiological relaxation and immune-function recovery through five-sense stimulation during forest therapy [1]. Several studies have shown that five-sense stimulation from forest therapy could improve anxiety and depression symptoms and stabilize the effects of autonomic dysfunction [22,23,24,25]. Second, forest oils, such as phytoncide, have several positive effects on physical and emotional health with respect to recovery from stressful conditions [18,19]. A phytoncide-exposure study in an urban environment showed an increase in the antioxidant effect [26]. Third, a regular life cycle with timed meals and simple exercise by taking walks through forest paths could help regulate the sleep–wake cycle and circadian rhythms with immune system recovery [27]. The influence of various forest therapeutic aspects has not been evaluated.

From the polysomnography results, we observed that the latency of sleep onset was shortened from 14.9 ± 19.8 min to 6.5 ± 6.7 min. However, this did not have a statistical significance. Further research is needed on randomized controlled trials to adjust the regular sleep–wake cycle, and to examine whether forest therapy itself can affect sleep quality.

Other variables, namely, the ratio of N1, N2, N3, and REM sleep and the Apnea–Hypopnea Index, did not show any differences between before and after forest therapy. No changes of sleep structure are more likely to be related to the length of the forest therapy than to the small sample size. AHI results suggest a mild-degree sleep apnea. One of the participants showed severe sleep apnea and started therapy with a continuous positive airway pressure.

This study has some limitations. First, the small number of patients participating in the forest therapy and the lack of a control group are the main limitation. Most of the patients lived in the city, so it was difficult for patients to physically travel to the forest facilities for therapy. Second, this study was not confined to a specific cancer, and it included patients at various stages of cancer and patients with various gastrointestinal tract cancers. Therefore, it is necessary to pay attention to the interpretation of the study results. Third, because of the variety of program elements, we did not know which specific element improved the quality of the patients’ sleep. Therefore, more specific studies are required to demonstrate the effectiveness of each element of the program. Fourth, this study could not prove the effect of improved sleep quality on cancer treatment. As mentioned earlier, several studies have shown that improving sleep conditions and quality has a positive effect on cancer treatment, but it was difficult to analyze the effect of sleep improvement on cancer treatment because of the short study period, the small number of participants, and their various cancers and cancer stages. Fifth, the Hawthorne effect is a type of reactivity in which individuals modify an aspect of their behavior in response to their awareness of being observed [28]. It also has the effect of some people working harder and performing better when they are participants in an experiment. Therefore, the results of the forest therapy could be related the psychological effects that sleep can improve patient wellbeing.

It was not easy to find participants who could participate because an immune/physical crisis could have occurred due to the underlying cancer. However, when we consider the impact of sleep disturbance on cancer patients and the positive results related to sleep quality after forest therapy, we suggest that these attempts to improve sleep disturbance symptoms in cancer patients should be continued.

## 5. Conclusions

The results showed that forest therapy is beneficial for the improvement of sleep quality with PSG, and especially the improvement of sleep efficiency in cancer patients. In addition, based on this study, it is expected that scientific research on the therapeutic effect of forests will be expanded, and governmental leadership for forest development is required so that many people can benefit from a forest available as a valuable public resource. Because this research was a pilot study, randomized controlled trials will be required to establish a strong basis for forest therapy.

## Figures and Tables

**Table 1:** Baseline characteristics.

Variables	
Age (Mean ± SD)	53.6 ± 5.8
Sex (M/F)	1/8
Height (Mean ± SD)	155.5 ± 5.7
Weight (Mean ± SD)	53.1 ± 10.2
BMI (Mean ± SD)	21.9 ± 3.4

BMI, Body mass index; SD, Standard deviation.

**Table 2:** Polysomnography findings.

Variables	Pre-Forest Therapy	Post-Forest Therapy	*p*
TST	367.2 ± 33.4	398.0 ± 33.8	0.020
Latency of sleep onset	14.9 ± 19.8	6.5 ± 6.7	0.293
Sleep efficiency	80.5 ± 7.1	88.4 ± 5.2	0.027
WASO	65.1 ± 34.6	53.5 ± 26.3	0.514
N1	22.1 ± 9.7	16.0 ± 6.6	0.084
N2	49.7 ± 7.1	54.1 ± 5.6	0.074
N3	11.9 ± 5.2	12.8 ± 5.8	0.543
REM	16.3 ± 6.4	17.1 ± 5.8	0.758
AHI	10.9 ± 4.1	13.4 ± 12.2	0.272
PLMi	8.9 ± 13.1	12.0 ± 19.9	0.524

TST, Total sleep time; WASO, Wake after sleep onset; N1, Non-REM sleep stage 1; N2, Non-REM sleep stage 2; N3, Non-REM sleep stage 3; REM, Rapid eye movement sleep; AHI, Apnea–Hypopnea Index; PLMi, Periodic Limb Movements index.

**Table 3:** Sleep questionnaire results.

Variables	Pre-Forest Therapy	Post-Forest Therapy	*p*
PSQI-K	8.0 ± 4.4	7.3 ± 4.9	0.299
STOP BANG	1.6 ± 1.1	1.5 ± 1.1	0.347
SSS	2.1 ± 1.2	2.0 ± 1.1	0.729
ESS	6.1 ± 4.9	6.9 ± 5.6	0.393
HADS_Anxiety	5.0 ± 4.0	4.9 ± 4.2	0.855
HADS_Depression	3.6 ± 3.2	3.2 ± 3.1	0.438
ISI	5.8 ± 4.6	6.3 ± 5.3	0.621

PSQI-K, Korean version of the Pittsburgh Sleep Quality Index; STOP BANG, STOP BANG sleep apnea questionnaire; SSS, Stanford Sleepiness Scale; ESS, Epworth Sleepiness Scale; HADS, Hospital Anxiety and Depression Scale; ISI, Insomnia Severity Index.
